# Photocatalysis for Renewable Energy Production Using PhotoFuelCells

**DOI:** 10.3390/molecules191219732

**Published:** 2014-11-27

**Authors:** Robert Michal, Stavroula Sfaelou, Panagiotis Lianos

**Affiliations:** 1 Department of Chemical Engineering, University of Patras, University Campus, Patras 26500, Greece; E-Mails: michal@nic.fns.uniba.sk (R.M.); sfaelou@upatras.gr (S.S.); 2 FORTH/ICE-HT, Stadiou Str., Platani, P.O. Box 1414, Patras 26504, Greece

**Keywords:** PhotoFuelCells, photoelectrochemical cells, solar energy conversion, quantum dot sensitizers, TiO_2_

## Abstract

The present work is a short review of our recent studies on PhotoFuelCells, that is, photoelectrochemical cells which consume a fuel to produce electricity or hydrogen, and presents some unpublished data concerning both electricity and hydrogen production. PhotoFuelCells have been constructed using nanoparticulate titania photoanodes and various cathode electrodes bearing a few different types of electrocatalyst. In the case where the cell functioned with an aerated cathode, the cathode electrode was made of carbon cloth carrying a carbon paste made of carbon black and dispersed Pt nanoparticles. When the cell was operated in the absence of oxygen, the electrocatalyst was deposited on an FTO slide using a special commercial carbon paste, which was again enriched with Pt nanoparticles. Mixing of Pt with carbon paste decreased the quantity of Pt necessary to act as electrocatalyst. PhotoFuelCells can produce electricity without bias and with relatively high open-circuit voltage when they function in the presence of fuel and with an aerated cathode. In that case, titania can be sensitized in the visible region by CdS quantum dots. In the present work, CdS was deposited by the SILAR method. Other metal chalcogenides are not functional as sensitizers because the combined photoanode in their presence does not have enough oxidative power to oxidize the fuel. Concerning hydrogen production, it was found that it is difficult to produce hydrogen in an alkaline environment even under bias, however, this is still possible if losses are minimized. One way to limit losses is to short-circuit anode and cathode electrode and put them close together. This is achieved in the “photoelectrocatalytic leaf”, which was presently demonstrated capable of producing hydrogen even in a strongly alkaline environment.

## 1. Introduction

Simultaneous conversion of solar energy into chemical energy and electricity is achieved by means of photoelectrochemical cells. A standard configuration for a photoelectrochemical cell involves a photoanode electrode carrying an n-type semiconductor photocatalyst, a counter electrode carrying an electrocatalyst and an electrolyte. Light is absorbed by the photocatalyst generating electron-hole pairs. Electrons are guided through an external circuit to the cathode (counter) electrode, where they take part in reduction reactions while holes are consumed through oxidation reactions. In regenerative solar cells, the electrolyte involves a redox couple, which is reduced at the counter electrode and oxidized at the photoanode electrode thus refilling holes and converting photon energy into electricity. Those cells act as photovoltaic devices. The present work will not deal with such cells but it will rather focus on photoelectrochemical procedures where both oxidation and reduction reactions involve consumption or production of chemical substances. In that case, holes are consumed by oxidizing an organic or inorganic substance, which acts as a sacrificial agent. Electrons are consumed by some standard reduction reactions, for example, water, oxygen or CO_2_ reduction. A less common configuration for a photoelectrochemical cell involves a photocathode electrode carrying a p-type photocatalyst while the anode electrode carries an oxidation electrocatalyst. The present work will deal with a standard cell employing a photoanode and n-type semiconductor photocatalyst. In both types of cells, the energy of photons and the chemical energy of the sacrificial agent, *i.e.*, of the “fuel”, are converted into electricity and/or stored in a useful form of chemical energy, for example, production of hydrogen. For this reason, photoelectrochemical cells operating by consumption of a fuel are called PhotoFuelCells (PFCs) [1,2].

The origins of photoelectrochemical cells can be traced back to the findings of Alexander-Edmond Becquerel in 1839, who reported for the first time the photovoltaic (in reality, the photoelectrochemical) conversion of light into electricity [3]. However, it was a work by Fujishima and Honda in 1972 [4] describing water splitting by using a TiO_2_ photoanode that set the foundations of modern photoelectrochemistry. After more than 40 years, the conception of a photoelectrochemical cell remains the same but the new materials and procedures employed for their construction has kept the field alive with ever increasing popularity. Photoanode electrodes are usually made by depositing nanoparticulate titania (np-TiO_2_) on transparent conductive glasses like Fluorine-doped Tin Oxide (FTO). The choice of a photocatalyst should be dictated by the position of its conduction and valence band, which define its oxidation and reduction capacities. A large variety of different semiconductor photocatalysts have been studied through the years and reported in several publications [5,6,7,8]. However, other factors finally become more important, like the ease of synthesis and deposition, the specific surface area of the obtained film, the abundance of materials and the effectiveness of their photocatalytic functionality. Thus nanoparticulate titania is the champion of all photocatalysts, despite of some known disadvantages. The most serious of these disadvantages is the fact that it only absorbs UV radiation. In order to utilize this photocatalyst and at the same time exploit visible light, np-TiO_2_ must be combined with appropriate sensitizers. The present work, among others, reviews our approach for the use of visible-light-absorbing sensitizers. A second main issue to face in the study of PFCs and of all photoelectrochemical cells is the nature of the electrocatalyst. Nanoparticulate Pt is acknowledged as the best electrocatalyst for both oxidation and reduction reactions. There are two reasons for this quality. Pt can be easily obtained in fine nanoparticles and it has the highest work function, thus becoming an electron sink, which can exchange charges with the active liquid phase. However, Pt is rare and costly and this discourages large scale applications. Alternative materials employed as electrocatalysts should be also deposited in nanoparticulate form providing high specific surface area and should be good conductors facilitating transfer of charges. The present work will shortly describe our approach for the employment of alternative reduction electrocatalysts. Finally, the third principal issue involved in the use of PFCs is the choice of the fuel. Any organic substance can play the role of fuel and be photocatalytically oxidized during PFC operation. In fact, one of the main assets of a PFC is its capacity to oxidize any organic substance and produce usable energy. Thus water soluble wastes or products of biomass can be used as fuel, offering the multiple environmental benefit of water cleaning, of using biomass that may be too costly to refine and of producing renewable energy. Small chain length alcohols have been used as model fuels to employ with PFCs. In the present work, we use ethanol as model organic fuel. On the contrary, the choice of inorganic fuels is very limited. Most published works use sulfur containing compounds, for example, a combination of Na_2_S with Na_2_SO_3_ [9]. The present work will not deal with inorganic sacrificial agents.

## 2. Model of PFC Operation

The PFC operation reactions are summarized in Table 1 and are detailed as follows: reactions in Table 1 are designed for ethanol, which is presently employed, as already said, as a model fuel. When UV light is shined on the photocatalyst (np-TiO_2_), it is excited generating e^−^—h^+^ pairs (reaction (I)). These photogenerated charges may partly recombine and dissipate their energy. Electrons escaping recombination may be partly retained in the presence of O_2_ leading to formation of superoxide radical and then to ^•^OH radicals, which add to the degradation capacity towards the organic fuel (reaction (II)). The majority of free electrons flow through the external circuit. Of course, in the absence of oxygen all electrons escaping recombination flow through the external circuit.

The type of reactions induced by photogenerated holes depend on solution pH and on the presence or absence of O_2_. In the absence of O_2_ and at low pH, holes mediate the generation of hydrogen ions H^+^ according to reaction III. Reaction III represents an overall scheme. In reality, the reaction proceeds by steps, where the following route usually prevails [2]:

Ethanol → acetaldehyde → acetic acid → CO_2_ + H_2_O (1)

**Table 1:** Typical reactions taking place in a PFC employing ethanol as model fuel.

Photoanode	
(1) Reactions induced by absorption of UV photons (radiation mainly absorbed by the majority species, i.e., np-TiO_2_) (I) ${\text{TiO}}_{2}\overset{h\nu }{\to }e^{-}+h^{+}$ Fate of the photogenerated electrons: Most electrons flow in the external circuit. Some may interact with O_2_: (II) ${\text{O}}_{2}\overset{+\;e^{-}}{\to }{\text{O}}_{2}^{•-}\overset{+\;{\text{H}}^{+}}{\to }{\text{HO}}_{2}^{•}\overset{+\;e^{-}}{\to }{\text{HO}}_{2}^{-}\overset{+\;{\text{H}}^{+}}{\to }{\text{H}}_{\text{2}}{\text{O}}_{\text{2}}\overset{+\;e^{-}}{\to }{\;}^{•}\text{OH}+{\text{OH}}^{-}$ Fate of the photogenerated holes C_2_H_5_OH + 3 H_2_O + 12 h^+^→ 2 CO_2_ + 12 H^+^ (low pH) (III) OH^−^ + h^+^→ OH^•^ and C_2_H_5_OH+12 OH^•^→ 2 CO_2_ + 9 H_2_O (high pH) (IV) Intermediate steps may involve interaction of the fuel with photogenerated holes in the valence band of titania or holes injected into the valence band of the sensitizer C_2_H_5_OH + 2 h^+^→ CH_3_CHO + 2 H^+^ or C_2_H_5_OH + h^+^→ C_2_H_5_O^•^ + H^+^ (V)	
(2) Reactions induced by absorption of Visible light (exclusive excitation of the photosensitizer) Excited electrons are rapidly injected into the conduction band of TiO_2_. Some may interact with O_2_ forming superoxide radical and following the scheme of reaction II. Most electrons flow through the external circuit. Holes at the valence band of the photosensitizer may go through reactions III–V depending of the nature of the photosensitizer Other schemes may also be possible	
**Cathode**	
*Inert environment (no O_2_ present)*	*Aerated electrolyte or cathode exposed to ambient air*
Low pH (potential: 0.00 V at pH = 0) 2 H^+^ + 2e^−^→ H_2_ (VI) High pH (potential: −0.83 V at pH = 14) 2 H_2_O + 2 e^−^→ H_2_ + 2 OH^−^ (VII)	Low pH (potential: 1.23 V at pH = 0) 2 H^+^ + ½ O_2_ + 2 e^−^→ H_2_O (VIII) High pH (potential: 0.40 V at pH = 14) H_2_O + ½ O_2_ + 2 e^−^→ 2 OH^−^ (IX)
**Overall Cell Reactions** (combination of anode and cathode reactions)	
absence of oxygen (ethanol reforming): C_2_H_5_OH + 3 H_2_O → 2 CO_2_ + 6 H_2_ (X) presence of oxygen (ethanol mineralization): C_2_H_5_OH + 3 O_2_→ 2 CO_2_ + 3 H_2_O (XI)	

Most of these intermediate reactions are endothermic. The Gibbs free energy change for reaction (III) is positive, therefore the overall balance is endergonic and is mediated by photogenerated holes. The same holds true for all reactions associated with chemical substances of the general composition C_x_H_y_O_z_, which are generalized by the following scheme [2]:

(2) $$C_{x}H_{y}O_{z}+(2x-z)H_{2}O+(4x+y-2z)h^{+}\to xCO_{2}+(4x+y-2z)H^{+}$$

At high pH, a lot of ^•^OH radicals are formed by interaction of OH^−^ with holes

(3) $$OH^{-}+h^{+}\to {\;}^{•}OH$$

therefore, photocatalytic degradation should be visualized with their participation. In reality, ethanol photodegradation still follows the route of Equation (1) through intermediate acetaldehyde formation, as it has been previously detected and analyzed [10]. However, the number of hydrogen ions should be limited at high pH or completely eliminated by interacting with OH^−^ and producing water. Thus at high pH the photocatalytic degradation of ethanol should be represented by reaction IV if Table 1 while a more general scheme equivalent to Equation (2) should be defined by the following equation:

(4) $$C_{x}H_{y}O_{z}+{\left(4x+y-2z\right)}^{•}OH\to xCO_{2}+(2x+y-z)H_{2}O$$

where we assume that H^+^ + OH^−^→ H_2_O and H_2_O + h^+^→ H^+^ + ^•^OH. In the presence of oxygen, alternative photocatalytic degradation routes may be activated, however, what is mainly affected by the presence of oxygen is the production of molecular hydrogen as will be seen in the next paragraph. We have previously found that the presence of O_2_ accelerates mineralization of ethanol [10,11] at the expense of the current flowing in the external circuit. Special attention must be paid to the second reaction V of Table 1, showing ethanol radical formation by direct interaction with one hole. This radical is unstable and by interaction with the photocatalyst it injects an electron into its conduction band. This is responsible for the so-called “Current Doubling” effect [2], which will be further discussed in Section 3.1.

Reactions VI–IX taking place at the cathode electrode, as seen in Table 1, are less complicated and are independent of what is the fuel or the photocatalyst. It is seen that in the absence of oxygen, hydrogen is produced by reduction of hydrogen ions at low pH or by reduction of water at high pH. In the presence of O_2_, reactions proceed with the participation of the latter. Of course, no hydrogen is produced in that case. The electrochemical potentials for the reduction reactions are given in Table 1. The variation of the potential with pH follows the general rule [8]:

(5) ΔV(Volts)=−0.059×ΔpH

**Figure 1:**
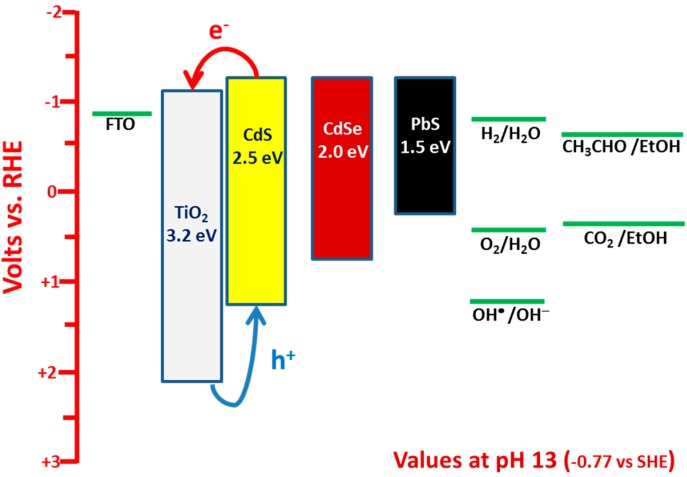
Approximate energy levels of a few semiconductors together with some characteristic redox potentials. The levels correspond to pH 13. They were negatively shifted with respect to the values at pH = 0, according to Equation (5). Thus at pH = 0 the potential of H_2_/H_2_O is zero. CdS, CdSe and PbS are shown as sensitizers by Fermi level alignment with TiO_2_. The band gap values correspond to quantum dot nanoparticles and are larger than in bulk semiconductors.

The values of these potentials define the conditions of PFC operation. When reactions VI–IX are combined with reactions III and IV they yield the characteristic schemes of cell operation, which are independent of the pH but distinguish themselves by the presence or not of oxygen. Thus electricity flows through the external circuit and hydrogen is produced in the absence of oxygen while in its presence only electricity is produced. These two overall operation schemes are represented by reactions X and XI of Table 1. The corresponding schemes for substances of the general composition C_x_H_y_O_z_ are given by:

(6) $$C_{x}H_{y}O_{z}+(2x-z)H_{2}O\to xCO_{2}+(2x+\frac{y}{2}-z)H_{2}$$

in the absence and by:

(7) $$C_{x}H_{y}O_{z}+(x+\frac{y}{4}-\frac{z}{2})O_{2}\to xCO_{2}+\frac{y}{2}H_{2}O$$

in the presence of oxygen. The Gibbs free energy change ΔG^0^ for Equation (7) has been calculated and listed in Table 2 for a few substances of the type C_x_H_y_O_z_. The overall balance is exergonic (ΔG^0^ < 0). The corresponding standard potential, was calculated by the following equation:

(8) $$E^{0}=\frac{\Delta G^{0}}{-\text{n}F}$$

where *n* is the number of electron moles involved in the reaction and F is the Faraday constant 96.486 kC·mol^−1^, while ΔG^0^ is given in kJ·mol^−1^. *n* and E^0^ are also listed in Table 2. The number of electrons (and holes) is equal to *n* = (4 x + y − 2 z), *i.e.*, equal to the number of holes involved in reactions Equations (2) and (4) or to 4 electrons per oxygen molecule involved in reaction Equation (7). Thus all potentials are positive and their range is limited between 1.12–1.21 V with respect to the Standard Hydrogen Electrode (SHE). The corresponding value for ethanol is +1.14 V. Figure 1 shows energy levels for some semiconductors and redox reactions at pH 13. Alkaline pH is chosen because, as it will be discussed later, PFCs usually operate at high pH. In the diagram of Figure 1, the potential for ethanol mineralization in the presence of oxygen is depicted as EtOH/CO_2_ level. Values in Figure 1 are referred to Reversible Hydrogen Electrode (RHE) to take account of the pH. Since pH = 13, it corresponds to a negative potential shift by 0.059 × 13 = 0.77 V, then the level of EtOH/CO_2_ in Figure 1 is placed at 1.14 − 0.77 = +0.37 V. Furthermore, reaction (V), which is the first step of ethanol oxidation, with ΔG^0^ equal to 34.8 kJ mol^−1^ and corresponding E^0^ = −0.18 V is placed in the diagram of Figure 1 at 0.18 − 0.77 = −0.59 V. The diagram of Figure 1 reveals that for holes found at the valence band level of titania, the oxidation power is high enough to carry out all listed reactions. However, in the presence of a sensitizer, holes are injected into the higher lying level of the sensitizer and then their oxidative power dramatically decreases. Thus all depicted sensitizers can oxidize ethanol to acetaldehyde, CdS and CdSe may catalyze ethanol mineralization and water oxidation but only CdS is oxidative enough to generate ^•^OH radicals. Direct scavenging of holes by ethanol means that ethanol molecules are adsorbed on the semiconductor, but it is easier to react with ^•^OH, which can take place in the liquid phase. Therefore, titania and CdS have higher chance to oxidize ethanol than CdSe and PbS. It is obvious that these interactions are fairly complicated. Obviously, mineralization of the fuel is encouraged by photocatalysts with high enough oxidation capacity.

**Table 2:** Calculated Gibbs free energy change and standard potential for reaction Equation (7) and for a few organic substances of the general composition C_x_H_y_O_z_.

Name	Chemical Composition	Number of Electrons	ΔG^0^(kJ·mol^−1^)	E^0^ (V)
Methanol	CH_3_OH	6	−702	1.21
Ethanol	C_2_H_5_OH	12	−1325	1.14
*n*-Propanol	C_3_H_7_OH	18	−1965	1.13
*n*-Butanol	C_4_H_9_OH	24	−2595	1.12
*n*-Pentanol	C_5_H_11_OH	30	−3249	1.12
Glycerol	C_3_H_8_O_3_	14	−1656	1.22
Sorbitol	C_6_H_14_O_6_	26	−3084	1.23
Acetic acid	CH_3_COOH	8	−873	1.13

If titania is combined with a sensitizer then visible light absorption is possible. When visible light is shined on the photoanode, only the photosensitizer is excited, since titania does not absorb in the visible. Electrons are rapidly injected into the conduction band of titania (*cf.* Figure 1) and follow the routes related to reaction Equation (II) of Table 1. The fate of the holes in the valence band of the sensitizer depends on the valence band potential, as already discussed. Photocatalytic degradation of alcohols using oxide photocatalysts has been extensively studied, however, rare data or no data at all exist for combined titania-QD photocatalysts. The reason is that such systems are thought to be unstable. This is not the case for certain semiconductor sensitizers like CdS, as has been shown in previous publications [12,13].

The conduction band level of nanocrystalline titania lies at about −0.2 to −0.3 V *vs.* SHE [5,6,7,8,14] or about −1 V *vs.* RHE (pH 13). Then the potential difference with the cathode is ideally no more than 0.3 V in the absence of oxygen (*cf.* Figure 1 and Table 1). This difference, taking into account the inevitable losses, is not strong enough to provide drive for cell operation. Consequently, a bias is necessary in that case, which can be applied either externally or chemically. If the anode is in an alkaline environment and the cathode in acidic environment a chemical forward bias is provided [15] according to Equation (5). When the cathode electrode is in an aerated environment, a potential difference of around 1.4 V is established between the anode and the cathode electrode as seen in Figure 1 and Table 1. Indeed, experimental data have repeatedly fulfilled this expectation [12,16]. PFCs then operating in the presence of oxygen produce high voltage and operate without bias, like solar cells. Obviously, in that case a PFC can be used as a device to produce electricity consuming organic wastes and being activated by absorption of light. Despite the fact that in the absence of oxygen a bias is necessary to run the cell, as already said, it is still possible to produce hydrogen by minimizing the losses. This has been achieved by minimizing the resistance of electron flow between anode and cathode electrodes. An example of a functional cell of this type will be presented in the present work. The following sections present a short review on experimental data obtained by the above described PFC types including some unpublished data on electricity and hydrogen production using PFCs.

## 3. Results and Discussion

### 3.1. Study of PFCs Operating in the Presence of Oxygen and Producing Electricity

**Figure 2:**
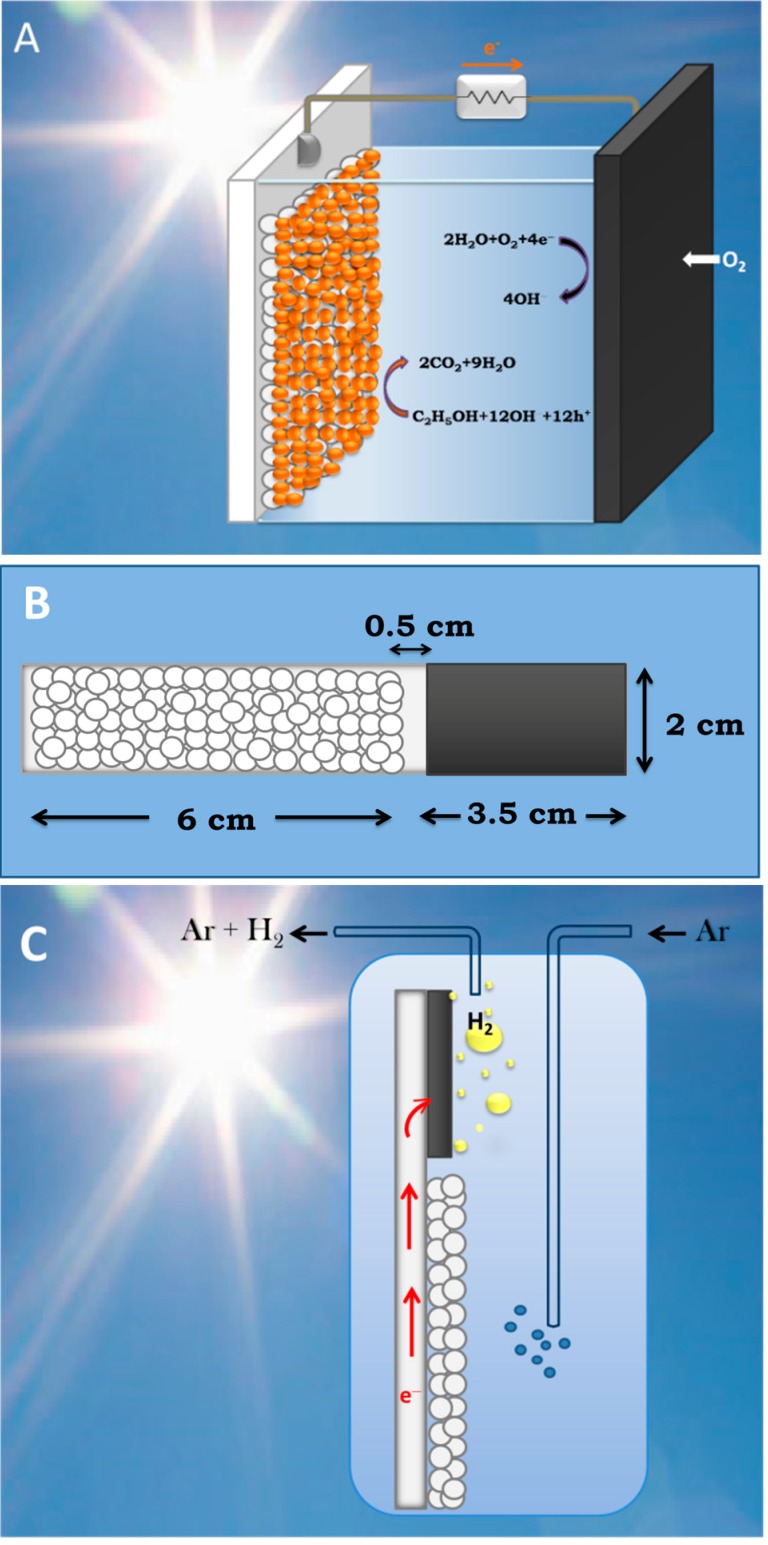
Design of reactors and electrodes: (**A**) Design of a PFC exclusively producing electricity; (**B**) Geometrical distribution of photocatalyst (small circles) and electrocatalyst (black area) on a “photoelectrocatalytic leaf”; and (**C**) Production of hydrogen using the “photoelectrocatalytic leaf”.

**Figure 3:**
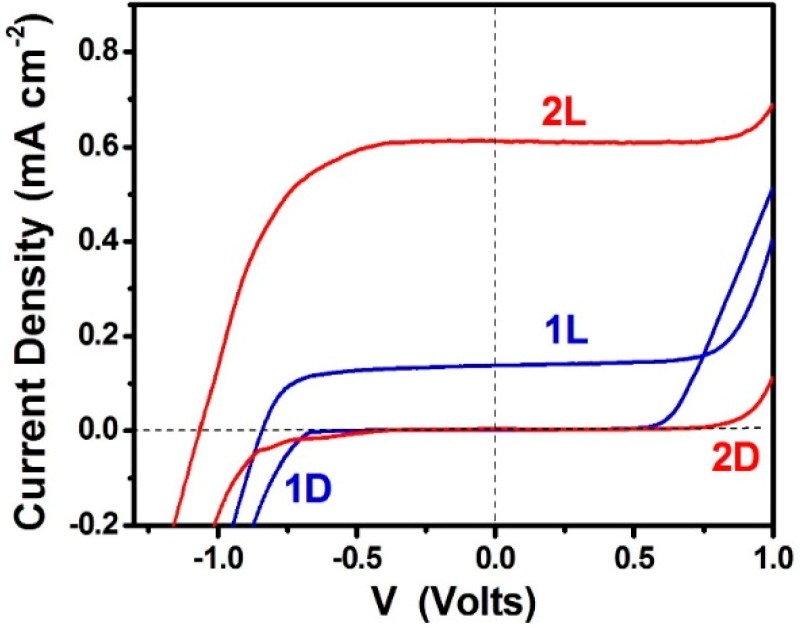
Current density-Voltage curves obtained with a PFC functioning in the presence of oxygen. The aqueous electrolyte always contained 0.5 mol·L^−1^ NaOH without (curve 1) or with (curve 2) 5 v % ethanol. L signifies irradiation (Light) and D dark. In both cases photoanode carried bare np-TiO_2_ film. The curves were traced in a 2-eletrode configuration.

**Figure 4:**
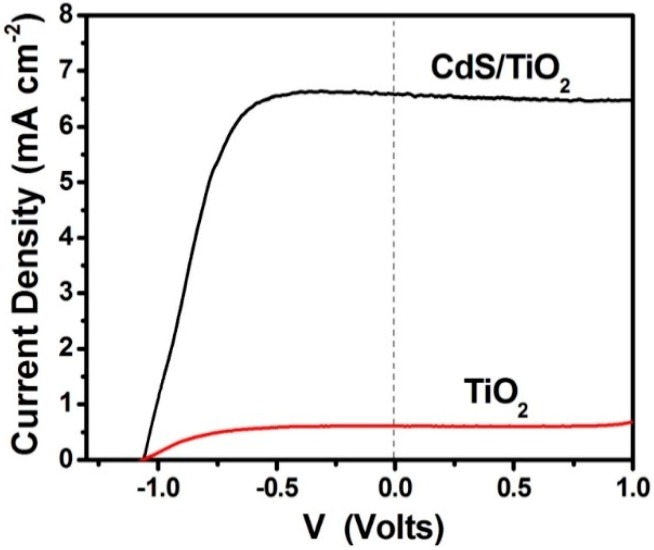
Current density-voltage curves obtained with a PFC functioning in the presence of oxygen. The aqueous electrolyte always contained 0.5 mol·L^−1^ NaOH and 5 v % ethanol. Both curves were obtained by illumination with simulated solar radiation. The lower curve corresponds to a photoanode carrying bare titania while the upper curve corresponds to a CdS-sensitized titania photoanode. The curves were traced in a 2-eletrode configuration.

PFCs operating in the presence of oxygen with the purpose of producing electricity by photocatalytically consuming an organic fuel are schematically represented by Figure 2A. The photoanode carries np-TiO_2_ photocatalyst with or without sensitizer. The cathode electrode is made of “air-breathing” carbon cloth on which a hydrophobic material (usually, carbon black and Teflon paste) is deposited. This hydrophobic paste prevents water leak through the carbon cloth. On this modified electrode a catalytic layer is deposited. The best catalyst is nanoparticulate Pt, as in the present case (see Experimental Section). However, satisfactory results were obtained in the past by employing carbon nanotubes or organic conductive polymers [17,18] as electrocatalysts. The fuel is added in the aqueous alkaline electrolyte. The cell can be also operated in the absence of fuel by oxidizing water, acting as purely water-splitting apparatus. Figure 3 shows JV characteristics obtained in a 2-electrode configuration using an air saturated PFC comprising a photoanode carrying bare titania and filled with NaOH electrolyte without fuel. The cell produced zero current in the dark but gave substantial current under illumination by a Xenon lamp, providing simulated solar radiation. Of course, in that case only the UV portion of the lamp is absorbed by the photocatalyst. In the presence of fuel (ethanol) both open-circuit voltage V_oc_ and short-circuit current density J_sc_ increased (curve 2 L of Figure 3). It is obvious that the presence of the fuel results in higher consumption of holes liberating more electrons. More electrons means higher current and, since the increase of electron population makes the conduction band of titania more negative, according to the discussion is Section 2, the potential difference between the anode and the cathode electrode increases, thus making V_oc_ higher. Indeed, in the absence of fuel J_sc_ and V_oc_ were 0.14 mA and 0.84 V, respectively but in its presence they became 0.61 mA and 1.03 V, respectively. However, part of the current recorded in the presence of fuel, may also come from “Current Doubling”, as it will be discussed below. The above results show that it is possible to produce electricity by consumption of a fuel using a PFC. Water itself can play the role of fuel but the current produced is lower and so is the voltage. The current can be much higher if a sensitizer is used and a greater portion of the solar radiation is absorbed. This is shown in Figure 4, where it is seen that J_sc_ increased more than tenfold when CdS quantum dots were deposited on the np-TiO_2_ film. Indeed, J_sc_ in that case became 6.6 mA. Interestingly, V_oc_ of the sensitized photoanode was the same as the non-sensitized one. This is expected. V_oc_ is approximately the difference between the conduction band level of nanocrystalline titania and the reduction level at the cathode electrode. In an alkaline environment, as in the present case, according to the discussion in Section 2, it is expected that V_oc_ may be as high as 1.4 V and it may be even higher in the presence of the fuel, which consumes holes and increases conduction band electronegativity, as already said. This value was never obtained due to inevitable losses. In addition, it is possible that reaction IX of Table 1 may be substituted by other reduction reactions with less positive potential, for example hydrogen peroxide formation (*cf.* [2]). Whatever is the case, the potential of the conduction band of titania defines the upper level. In the presence of CdS, if the latter acted as an independent semiconductor, this upper level would have been modified (*cf.* Figure 1). Because CdS acts as a sensitizer, its excited electrons are injected into the conduction band of titania, therefore, the latter defines the upper level. In fact the non-variation of V_oc_ in the presence of CdS is an index of sensitization.

It has been thus shown that CdS is an efficient sensitizer of titania in PFCs, applicable in aqueous alkaline environments. How stable is this sensitizer? A sensitizer remains stable as long as the photogenerated holes are consumed thus preventing sensitizer photo-corrosion by self-oxidation. This matter has been treated in previous publications [12,13]. It has indeed been found that if the oxidative power of the combined semiconductor-sensitizer is high enough, in order to be capable of producing hydroxyl radicals (*cf.* Figure 1), which are the main scavengers of photogenerated holes, then hole scavenging is ensured and the sensitizer is preserved. If the oxidative power is not sufficiently high, *i.e*., the valence band is not positive enough, as in the case of CdSe and PbS, then hole scavenging will rely on less efficient routes and the sensitizer becomes more vulnerable to oxidation. The best case certified by the previous findings is sensitization by moderately sized CdS quantum dots [13], as in the present case.

**Figure 5:**
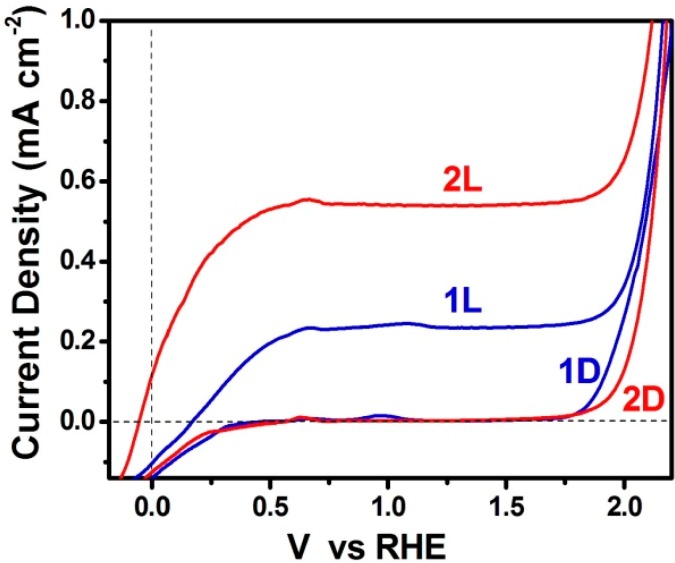
Current density-Voltage curves obtained with a PFC functioning in the presence of oxygen. The aqueous electrolyte always contained 0.5 mol·L^−1^ NaOH without (curve 1) or with (curve 2) 5 v % ethanol. L signifies irradiation (Light) and D dark. In both cases photoanode carried bare np-TiO_2_ film. The curves were traced in a 3-electrode configuration using Ag/AgCl as reference electrode. The curves are presented *vs.* RHE by adding 0.97 V to the recorded potential.

When the cell functions in the absence of organic fuel, photogenerated holes oxidize water. The data of Figure 3 show that it is easier to oxidize an organic fuel than to oxidize water. This is because organic substances can be oxidized by interaction with hydroxyl radicals, which, as already said, are efficient hole scavengers. Water cannot be easily oxidized because it takes two unit charges to split water, while an organic molecule can be oxidized by single steps, similar to that shown by the second reaction V of Table 1. This difficulty in oxidizing water precludes the employment of a sensitizer in the absence of a sacrificial agent. Thus CdS-sensitized photoanode was rapidly decomposed when used in the absence of ethanol [12]. Therefore, in the absence of fuel, titania must function alone without sensitizer. In this respect, it is interesting to also inspect the data presented in Figure 5, where current-voltage curves are plot in a 3-electrode configuration. It is seen that the saturation current in the presence of ethanol is to a rough approximation the double of the current in its absence. This finding calls for the “current doubling” effect frequently observed with such systems [2,19]. Current doubling is attributed to the photocatalytic formation of unstable organic radicals, as in the second reaction V of Table 1. The radical interacts with titania injecting an electron into its conduction band, according to the following reactions:

CH_3_CH_2_OH + h^+^ → CH_3_CH_2_O^•^ + H^+^ (9)

CH_3_CH_2_O^•^ → CH_3_CHO + H^+^ + e^−^ (10)

Thus for each photon absorbed, there are two electrons created in the conduction band, one photogenerated and one injected by the radical. This doubles the current, hence the term current doubling. We have not observed current doubling systematically [2,16] and it was not detected by the 2-electrode plots seen in Figure 3. We believe that current doubling is frequently shadowed by the normal photocatalytic mineralization route. That is, if the current recorded in the absence of fuel is low, the current observed in its presence may appear several times higher and not just the double [2,16]. The current observed in the absence of fuel may be small because of electron-hole recombination. In the presence of fuel, consumption of holes decreases recombination thus liberating more electrons and increasing current. Therefore, increase of current in the presence of an organic sacrificial agent may both come from the current doubling effect and from the decrease of electron-hole recombination. current doubling is surely responsible for unusually high currents. We have, for example, sometimes observed photon to electron conversion efficiencies, which are higher than 100% [12,20]. Even in the present case, CdS-sensitized photoanode gave high current, as seen in Figure 4, just above the theoretical limit. Indeed, for sensitizer band gap equal to 2.5 eV (496 nm) the theoretical current density expected for 100% efficiency is 6.3 mA·cm^−2^. The presently recorded short-circuit current density in Figure 4 was 6.6 mA·cm^−2^. Such unexpected high current can only be justified if we accept that current doubling does take place during PFC operation.

### 3.2. Use of PFCs for Hydrogen Production

PFCs operating in an inert environment, *i.e*., in the absence of oxygen, can produce hydrogen at the cathode electrode according to reaction VI or VII of Table 1. This has been verified in the past by using various cell configurations. As already analyzed in Section 2, a bias is necessary in order to produce a measurable quantity of hydrogen. Indeed, by using a two compartment cell separated by an ion transfer membrane, it is possible to operate the photoanode in an alkaline electrolyte and the cathode electrode in an acidic electrolyte. This introduces a chemical forward bias in the cell according to Equation (5). In addition, the acidic environment of the cathode electrode provides a reservoir of H^+^ ions, which can be reduced to generate hydrogen molecules. Such cells have been previously studied and reported [15,21,22]. Replacement of the consumed hydrogen ions is supposed to be made by hydrogen ions produced during oxidation of the fuel. However, since the photoanode is immersed in an alkaline electrolyte, these hydrogen ions would have to struggle to survive against interaction with hydroxyl ions. For this reason, such cells do not function for a long period of time unless a continuous supply of acid is assured.

Another possibility is to use a one-compartment cell filled with a single (alkaline) electrolyte and apply an electric forward bias. We have reported this case in a previous publication [23]. Hydrogen was indeed produced but the quantity was disappointingly low. Production of hydrogen in an alkaline environment is foreseen by reaction VII of Table 1. However, this reaction does not take into account losses and the fact that it is hard for hydrogen ions to survive in a highly alkaline environment. Surprisingly, even though, there is a lot of work on water splitting this issue of the employed electrolyte is not sufficiently studied and it calls for further investigations. In any case, it is necessary to limit losses in order to produce a sufficient quantity of hydrogen by using PFCs functioning in an alkaline environment.

### 3.3. Short-Circuiting Anode and Cathode Electrode for Hydrogen Production in an Alkaline PFC. The “Photoelectrocatalytic Leaf”

**Figure 6:**
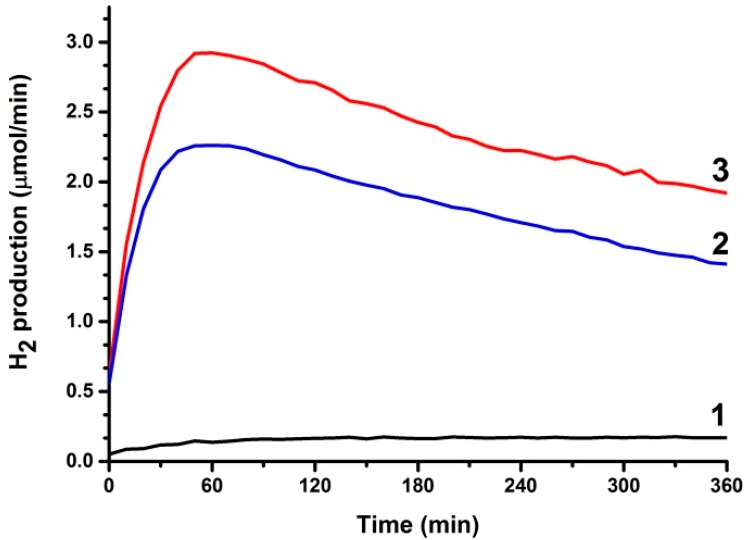
Hydrogen production rate using the “photoelectrocatalytic leaf” (short-circuited electrodes) of Figure 2B,C. The photoanode was made of np-TiO_2_ while the electrocatalyst was made of: (1) 4 mg of Elcocarb alone; (2) 0.6 mg Pt alone and (3) 0.08 mg of Pt dispersed in 4 mg of Elcocarb. The alkaline electrolyte contained 5 v % ethanol.

**Figure 7:**
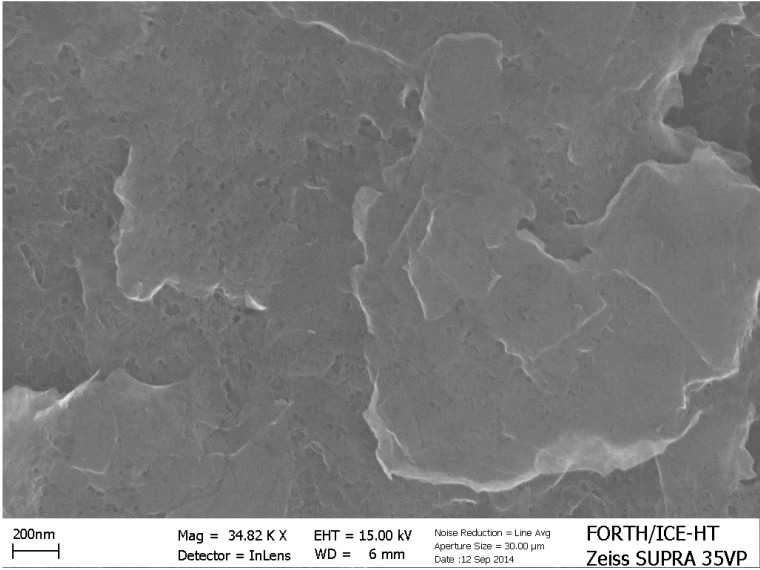
FESEM image of the Elcocarb film. The scale bar is 200 nm.

In the previous subsection it was stated that it is necessary to keep losses to a minimum in order to detect hydrogen production by a PFC in an alkaline environment. Alkaline environment is preferred because it offers a higher supply of hydroxyl radicals and enhances the photocatalytic capacity of titania, as analyzed in Section 2. One way to limit losses is to short-circuit anode and cathode electrode facilitating electron transfer from anode to cathode. This is usually done by depositing photocatalyst and electrocatalyst on the two sides of a metal support [21,22]. A more practical configuration is presented in the present work by the “photoelectrocatalytic leaf” of Figures 2B,C, which supports oxidation and reduction reactions side by side, like in natural photosynthesis. By lying next to each other, the distance between anode and cathode is decreased. Thus the route that ionic species have to run within the electrolyte is also decreased. In the present work, we present an easy to prepare and functional electrode using commercial materials deposited on a single FTO slide. As described in the Experimental section and depicted in Figure 2B, the photocatalyst covers an area of 6 cm × 2 cm and the electrocatalyst an area of 3.5 cm × 2 cm. Three possibilities of electrocatalyst have been tested (see Section 4.3). Pt casted directly on FTO, a commercial carbon paste called Elcocarb and Pt mixed with Elcocarb. All three were functional in producing molecular hydrogen, as can be seen in Figure 6. The curves of Figure 6 are structured in three parts: a rising part, which corresponds to hydrogen building up in the reactor and the tubing; the peak rate, which shows the relative efficiency of each electrocatalyst; and the falling part, showing exhaustion of the fuel. It is seen that Elcocarb paste alone gave very low hydrogen production rate. Pt alone gave much higher rate but the highest was obtained with the Pt-enriched Elcocarb paste. It must be underlined at this point that the quantity of Pt alone (0.6 mg) was much larger than the quantity of Pt mixed with Elcocarb paste (0.08 mg). Therefore, combination of these materials offers a very important advantage. As seen by its FESEM image shown in Figure 7, Elcocarb film is made of a nanostructured material that apparently provides a means for fine distribution of Pt nanoparticles. Thus much smaller quantity of platinum has a better electrocatalytic effect than pure Pt, which apparently loses its properties by aggregation.

The present “photoelectrocatalytic leaf” is one step apart from the combined np-TiO_2_-Pt photocatalyst and the photocatalytic hydrogen production, which is the most popular and the most efficient route for hydrogen production by using photocatalysts. In that case, Pt (or other noble metal) nanoparticles are deposited on titania nanoparticles and thus each combined particle is a nano-size device capable of oxidizing the fuel and at the same time of reducing hydrogen ions to molecular hydrogen. In that case, no electrolyte is necessary to make the system function and thus the above pH-associated issues become less important. Photocatalytic hydrogen production is very popular and it has been treated in hundreds of publications [22,24,25,26,27]. What interest could then be ascribed to the above “photoelectrocatalytic leaf”? The present data show that by separating the photocatalyst from the electrocatalyst, it is possible to employ an alternative material for the latter and avoid the use of a rare metal like Pt, or at least reduce its quantity, as in the present case.

## 4. Experimental Section

### 4.1. Materials

Unless otherwise indicated, reagents were obtained from Aldrich (Taufkirchen, Germany) and were used as received. Commercial nanocrystalline titania Degussa P25 (specific surface area 50 m^2^/g) was used in all cell constructions and Millipore (Merck-Millipore, Darmstadt, Germany) water was used in all experiments. SnO_2_:F transparent conductive electrodes (FTO, Resistance 8 Ω/square) were purchased from Pilkington (Toledo, OH, USA).

### 4.2. Preparation of np-TiO_2_ Films and Deposition of CdS by the SILAR Method

Nanoparticulate titania (np-TiO_2_) films were deposited on FTO transparent electrodes by the following procedure: a FTO glass was cut in the appropriate dimensions and was carefully cleaned first with soap and then by sonication in isopropanol, water and acetone. A thin layer of compact titania was first sprayed over a patterned area by using 0.2 mol·L^−1^ diisopropoxytitanium bis(acetylacetonate) solution in ethanol and was calcined at 500 °C. Deposition of this bottom compact layer is a common practice with nanocrystalline titania photoanodes, since it enhances attachment of the top thick film, prevents short circuits and facilitates electron flow towards the electrode. On the top of this compact film, we applied a titania paste made of P25 nanoparticles by doctor blading. The film was calcined up to 550 °C at a rate of 20 °C/min. The final thickness of the film, as measured by SEM, was approximately 10 µm. The geometrical area of the film varied according to the particular application. CdS was deposited by Successive Ionic Layer Adsorption and Reaction (SILAR) [12,13,28]. 10 SILAR cycles were applied by using Cd(NO_3_)_2_ as Cd^2+^ and Na_2_S as S^2−^ precursor. In all cases, we used 0.1 mol·L^−1^ aqueous solutions for both cations and anions. After SILAR deposition and final washing with tripled distilled water, the films were dried in an oven at 100 °C and were ready for use.

### 4.3. Construction of the Counter Electrode

Experimental data obtained for the purpose of the present work involve the following counter electrode constructions. Electrodes allowing O_2_ diffusion for the case of oxygen saturated cell operation were based on commercial carbon cloth, which was functionalized with carbon black and Pt similarly to previous publications [12,13,16]. A hydrophobic layer was first applied as follows: carbon black (0.246 g) was mixed with distilled water (8 mL) by vigorous mixing in a mixer (about 2400 r.p.m.) until it became a viscous paste. This paste was further mixed with polytetrafluorethylene (Teflon 60% wt. dispersion in water, 0.088 mL) and then applied on a carbon cloth cut to the necessary dimensions. This has been achieved by first spreading the paste with a spatula, preheating for a few minutes at 80 °C and finally heating also for a few minutes in an oven at 340 °C. Subsequently, the catalytic layer was prepared as follows: 1 g of Pt-carbon black electrocatalyst (30% on Vulcan XC72, Cabot, Leuven, Belgium) was mixed with Nafion perfluorinated resin (5 wt. % solution in lower aliphatic alcohols and water, 8 g) and a solution made of 7.5 g H_2_O and 7.5 g isopropanol (15 g). The mixture was ultrasonically homogenized and then applied on the previously prepared carbon cloth bearing carbon black. The electrode was then heated at 80 °C for 30 min and the procedure was repeated as many times as necessary to load about 0.5 mg of Pt/cm^2^. The thus prepared Pt-carbon black/carbon-cloth (Pt/CC) electrode was ready for use. Its dimensions were 2.25 cm^2^ (1.5 cm × 1.5 cm). The above porous carbon cloth electrode allows contact with air while the deposited hydrophobic materials prevent water leak.

For the purpose of hydrogen production the counter electrode was based on an FTO glass. In fact, in that case photoanode and cathode were patterned on the same FTO slide, spatially separated as shown in Figure 2B,C. One part was covered with photocatalyst while the other part was covered with the electrocatalyst. In this way the electrons could be directly transferred from the photocatalyst to electrocatalyst through the FTO layer without wiring, thus minimizing losses. This is a “photoelectrocatalytic leaf” similar to the “quasi-artificial leaf” described in [29] and also previously presented by us [30,31]. The photocatalyst was deposited by the procedure described in Section 3.2. The electrocatalyst was deposited by using the following alternatives: (1) A solution of Diamminedinitritoplatinum (II) in ethanol was cast on warm FTO so that the solvent evaporated and the remaining material formed a dark film. It was annealed at 450 °C forming a film of clustered Pt nanoparticles. The total quantity of Pt in the film was calculated to be 0.6 mg; (2) A commercial carbon paste named Elcocarb C/SP (Solaronix, Aubonne, Switzerland), was applied on FTO by doctor blading and was annealed at 450 °C. This paste forms a uniform and very stable film. Its quantity was 4 mg; (3) Elcocarb was mixed with Pt. The mixed material was again applied by doctor blading and was annealed at 450 °C. The quantity of Elcocarb was again 4 mg and it contained 2 wt % (0.08 mg) of Pt. Therefore, in the mixed Pt-Elcocarb film, the quantity of Pt was 7.5 times lower than when Pt was cast alone.

### 4.4. Device (Reactor) Construction

Two reactors were used for the purposes of the present work. When PFC was employed as a device functioning in the presence of oxygen to produce electricity, it was made of a Plexiglas body with the two electrodes facing each other at a distance of 5 mm. As depicted in Figure 2A, the photoanode electrode played the role of cell window while the opposite side was closed with the Pt/CC electrode. The quantity of the electrolyte was 10 mL and it contained 0.5 mol·L^−1^ NaOH with or without 5 v % ethanol. The active area of the photoanode was 1 cm × 1 cm = 1 cm^2^ and that of the counter electrode 1.5 cm × 1.5 cm = 2.25 cm^2^. In the case of hydrogen production using the electrode of Figure 2B,C, the reactor was a Pyrex cylinder containing 100 mL of an aqueous solution of 0.5 mol·L^−1^ NaOH and 5 v % ethanol. The double electrode of Figure 2B,C was completely immersed in the electrolyte and was placed in an upright position. The cylinder was equipped with fittings allowing Ar gas to flow through the electrolyte. The latter was deoxygenated for 20 min by a vigorous flow of Ar and then the flow continued at a controlled rate of 20 mL·min^−1^. Hydrogen production was monitored on-line by a gas chromatographer. Both reactors were illuminated by simulated solar light set at around 100 mW·cm^−2^. When titania was alone without sensitizer it was excited by the UV portion of the incident radiation.

### 4.5. Measurements

Current-voltage curves were obtained with the help of an Autolab PGSTAT128N potentiostat (Metrohm-Autolab, Utrecht, Holland). They were all traced at a rate of 5 mV·s^−1^. Ag/AgCl electrode was used as reference, when necessary, but corresponding plots were presented *vs.* Reversible Hydrogen Electrode (RHE) by adding 0.97 V to the recorded voltage values (*i.e*., +0.2 V for the potential of Ag/AgCl *vs.* SHE + 0.059 × 13, where 13 is the corresponding value of the pH). Hydrogen was detected on line by using a SRI 8610C gas chromatograph (Torrance, CA, USA). Calibration of the chromatograph signal was accomplished by comparison with a standard of 0.25% H_2_ in Ar. FESEM images were obtained with a Zeiss SUPRA 35VP (Oberkochen, Germany).

## 5. Conclusions

The present work has examined PhotoFuelCells (PFCs) as devices which can convert solar light to electricity or chemical energy, for example hydrogen, at the same time consuming a fuel, which can be a water waste or a water pollutant. PFCs thus offer the double benefit of renewable energy production and environmental remediation. PFCs can function in the presence of oxygen producing electricity with a high open-circuit voltage. Photoanodes carrying nanoparticulate titania can be sensitized in the Visible by quantum dot metal sulfide semiconductor sensitizers. The choice of sensitizer is limited in the case of PFCs since the combined titania-sensitizer photocatalyst must retain its oxidative power to oxidize the fuel. Only moderately sized CdS nanoparticles have been so far demonstrated to be efficient and stable sensitizers of nanoparticulate titania. Titania alone can oxidize water producing a rather low current, which increases in the presence of a fuel. A cell can run alone without any bias just by shining light, if the cathode is aerated. If it is necessary to produce hydrogen, that is, in the absence of oxygen, a bias must be applied. A chemical bias does lead to hydrogen production but it necessitates a continuous supply of acid. In an alkaline environment it is difficult to produce hydrogen even under bias because of the scarcity of hydrogen ions. It is still possible to produce hydrogen in an alkaline environment by minimizing losses and this was achieved by the “photoelectrocatalytic leaf” where anode and cathode electrode were short-circuited by being deposited on the same FTO slide.
